# Characteristics of Nutraceutical Chewing Candy Formulations Based on Fermented Milk Permeate, Psyllium Husk, and Apple By-Products

**DOI:** 10.3390/foods10040777

**Published:** 2021-04-05

**Authors:** Egle Zokaityte, Karolina Siriakovaite, Vytaute Starkute, Paulina Zavistanaviciute, Vita Lele, Erika Mozuriene, Dovile Klupsaite, Pranas Viskelis, Romas Ruibys, Raquel P. F. Guiné, Elena Bartkiene

**Affiliations:** 1 Department of Food Safety and Quality, Veterinary Academy, Lithuanian University of Health Sciences, Tilzes Str. 18, LT-47181 Kaunas, Lithuania; karolina.siriakovaite@stud.lsmu.lt (K.S.); vytaute.starkute@lsmuni.lt (V.S.); paulina.zavistanaviciute@lsmuni.lt (P.Z.); vita.lele@lsmuni.lt (V.L.); erika.mozuriene@lsmuni.lt (E.M.); elena.bartkiene@lsmuni.lt (E.B.); 2 Institute of Animal Rearing Technologies, Faculty of Animal Sciences, Lithuanian University of Health Sciences, Tilzes Str. 18, LT-47181 Kaunas, Lithuania; dovile.klupsaite@lsmuni.lt; 3 Institute of Horticulture, Lithuanian Research Centre for Agriculture and Forestry, Kauno Str. 30, LT-54333 Babtai, Lithuania; biochem@lsdi.lt; 4 Institute of Agricultural and Food Sciences, Agriculture Academy, Vytautas Magnus University, K. Donelaicio Str. 58, LT-44244 Kaunas, Lithuania; romas.ruibys@vdu.lt; 5 CERNAS Research Centre, Polytechnic Institute of Viseu, 3504-510 Viseu, Portugal

**Keywords:** nutraceutical chewing candy, overall acceptability, emotions induced in consumers, milk permeate, psyllium husk, apple by-products, antioxidant properties

## Abstract

The aim of this study was to develop nutraceutical chewing candy (CCN) formulations based on fermented milk permeate (MP) (source of galactooligosaccharides (GOS) and viable lactic acid bacteria (LAB)), psyllium husk (source of desirable hydrocolloids), and apple by-products (source of phenolic compounds). For CCN preparation, gelatin (Gel) and agar were tested; also, to provide CCN prepared using agar with a desirable hard texture, citric acid (cit) was changed to ascorbic acid. To select the optimal quantities of the ingredients, overall acceptability (OA) and emotions (EMs) induced in consumers by different CCN formulations were evaluated. Furthermore, viable LAB count during storage, texture, colour, and antioxidant characteristics were analysed. The highest OA (score 8.5) was shown for samples consisting of MP, psyllium husk (Ph), apple by-products (App), cit and xylitol (Xy); a very strong correlation was found between OA and the EM “happy” (*r* = 0.907**). After 14 days of storage, Gel+MP+Ph+App+cit samples showed a LAB count higher than 6.0 log_10_ CFU g^−1^; however, better antioxidant properties were found for the CCN prepared with agar. Finally, it can be stated that fermented MP, Ph, and App can be used for preparation of added-value CCN in a sustainable manner, and the recommended formulation is Gel+ MP+Ph+App+cit+Xy.

## 1. Introduction

The circular economy has many challenges, of which the most important is the need for attractive technologies for the effective recycling of by-products. Most of the technological solutions for recycling by-products (e.g., extraction) have limitations (use of additional chemicals, a non-desirable residue in the end product, and extracts can be concentrated not just in desirable but also toxic compounds, etc.). Moreover, it is difficult to integrate complex equipment into conventional industrial systems: they need specific qualified professionals for process realization, etc. For these reasons, whole by-product valorization technologies have become very attractive.

One by-product from the dairy industry is milk permeate (MP); however, studies about MP valorization are very limited. This by-product is obtained during the process of milk protein preparation, and contains milk sugar (lactose), minerals and serum proteins, and its bacterial contamination is very low. However, the pH of MP is similar to that of milk, and its sensory properties are not acceptable to consumers. It was reported, although MP cannot be directly suggested as a high-value food product by itself, that it is possible to improve its characteristics by applying fermentation with selected lactic acid bacteria (LAB) strains [1]. During the fermentation process, selected LAB convert lactose into galactooligosaccharides (GOS), which are considered prebiotic compounds. Moreover, fermented MP possesses desirable antimicrobial properties against various pathogenic strains [1].

The functional value of fermented MP can be improved by the addition of other natural ingredients, e.g., by-products from apple juice production. The apple juice industry generates a large amount of by-products [2], which have a high content of cell wall polysaccharides and various phenolic compounds [3,4]. Apple by-products possess antimicrobial properties and can inhibit a variety of pathogenic strains [5]. Till now, apple processing by-products have been used in very small amounts by recovering them into commercially feasible products [3,6]. However, demand for natural food ingredients is growing and many efforts have been put forth for sustainable use of bioresources, including apple by-products.

Psyllium husk is a source of natural hydrocolloids [7]. The plants from which it is obtained are well known for their positive health effects [8], and in 2012 the Food and Drug Administration (FDA) reported the positive effect of psyllium husk soluble fibre on reducing the risk of coronary heart disease [9]. Psyllium husk arabinoxylans are resistant to degradation in the gut [10]; they have prebiotic properties, which leads to an increase in desirable microorganisms in the digestive tract and a decrease in pathogenic bacteria [11,12]. Moreover, psyllium husk has unique gelling and good sensory properties, which are both desirable for food ingredients [13]. In addition, psyllium husk is a low-cost, biodegradable, and eco-friendly material [14,15].

Finally, we hypothesized that fermented MP, psyllium husk, and apple by-products can be included in nutraceutical chewing candy (CCN) formulas as sustainable and functional ingredients. To select the best formulations, different quantities of psyllium husk and apple by-products were tested for CCN formulations. In addition to standard overall acceptability (OA) methods, we used an emotion (EM) intensity scanning technique (FaceReader software), as we hypothesized that the implicit emotional responses revealed through facial expressions could indicate the interaction of consumers with products in a more sensitive manner, because EMs have a significant role in the comprehension of food preferences and consumer acceptability [16].

The aim of this study was to develop CCN formulations based on fermented MP, psyllium husk, and apple by-products, as sustainable and functional ingredients, and to select the best CCN formulation according to the product’s OA, the EMs it induces, and its microbiological and physicochemical characteristics. In addition, two texture-forming agents (gelatin and agar) were tested for CCN preparation.

## 2. Materials and Methods

### 2.1. Materials Used for CCN Preparation

For different CCN formulations, fermented MP, vacuum-dried apple by-products (App), psyllium husk powder (Ph), gelatin (Gel), agar, citric acid (cit), ascorbic acid (AA), and xylitol (Xy) were used (Table 1).

Fresh MP was obtained from the “Pienas LT” agricultural cooperative (Biruliskes, Lithuania), and stored at −18 °C before use. It was reported that the highest concentration of GOS and the most effective antimicrobial properties of MP can be obtained when *Pediococcus acidilactici* strain LUHS29 is used for fermentation of MP [1]. Characteristics of the biomodified MP used in this study for preparation of CCN are shown in supplementary file S1.

Vacuum dried apple (variety “Auksis”) by-products were received from the Institute of Horticulture, Lithuanian Research Centre for Agriculture and Forestry (Babtai, Kaunas distr., Lithuania) in 2020 [5]. It was reported that the apple (variety “Auksis”) by-products possess antimicrobial properties against *Bacillus cereus*, *Streptococcus epidermis*, *Staphylococcus haemolyticus*, and *Pasteurella multocida* (Supplementary file S2) [5].

Powder of Ph was obtained from Livin (Kaunas, Lithuania) in 2020.

Agar powder (*Gelidium sesquipedale* algae; Rapunzel, Germany) and gelatin (Klingai, Kaunas, Lithuania) were used for CCN formation. Furthermore, Xy (Natur Hurtig, Nuremberg, Germany), cit (Sanitex, Kaunas, Lithuania), and AA (Camelia pharmacy network, Kaunas, Lithuania) were tested for CCN preparation.

### 2.2. CCN Preparation

**Figure 1:**
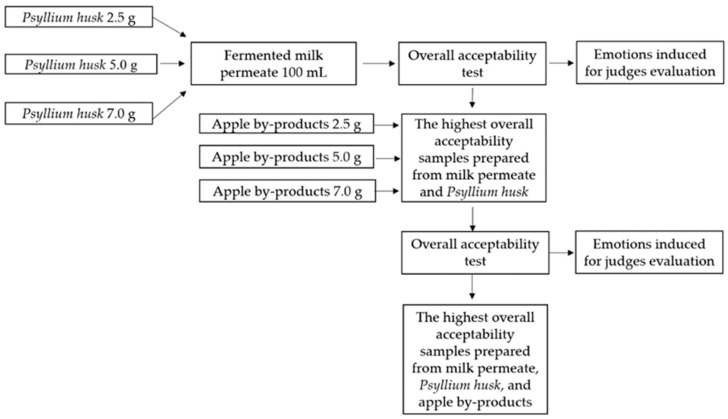
Scheme for selecting psyllium husk (Ph) and apple by-products for preparation of nutraceutical chewing candy (CCN).

First of all, the maximum quantities of Ph and, hereafter, apple by-products, according to the OA and EMs induced in consumers, were selected for CCN preparation (Figure 1). To select the optimal quantities, three different quantities of Ph and apple by-products were tested (2.5, 5.0, and 7.0 g in 100 mL of MP).

During the second step, the selected masses of MP, Ph, and apple by-products were used for preparation of CCN. CCN recipes are presented in Table 1.

For preparation of CCN with agar, firstly, agar powder was soaked in water for 30 min, and then melted by heating for 5 min; then, other ingredients were added and mixed under boiling conditions. The mixture obtained was further heated to 103 ± 2 °C under stirring.

For preparation of CCN with Gel, firstly, Gel powder was soaked in water for 30 min, and then melted at 80 ± 2 °C; then, other ingredients were added and mixed.

After mixing, the mass obtained (both that prepared with agar and prepared with Gel) was poured into a mould, and CCN were dried at 22–24 °C for 24 h to get a hard gel form.

Prepared CCN were analysed further, by evaluating their OA, EMs induced in consumers, colour coordinates, total phenolic compound (TPC) content, antioxidant activity, and LAB count during storage.

### 2.3. Evaluation of Overall Acceptability (OA) of CCN and Emotions (EMs) Induced in Consumers

The OA of the starter mixtures and prepared CCN was established by 50 judges, according to ISO method 8586-1 [17], using a 10-point scale ranging from 0 (“dislike extremely”) to 10 (“like extremely”). Similarly, the prepared CCN was tested by applying FaceReader 8.0 software (Noldus Information Technology, Wageningen, The Netherlands), scaling eight EM patterns (neutral, happy, sad, angry, surprised, scared, disgusted, contempt) and valence (scores ranged from −1 to 1) according to the procedure described by Bartkiene et al. [18].

### 2.4. Analysis of CCN Colour Characteristics and Texture

The colour coordinates (L*, a*, b*) were assessed using a CIELAB system (Chromameter CR-400, Konica Minolta, Tokyo, Japan).

Texture was evaluated using a Brookfield CT-3 Texture Analyser (Middleboro).

### 2.5. Determination of Viable LAB Count in CCN Formulations during Storage

For evaluation of the viable LAB count procedure described by Zokaityte et al. [1] was used. Viability of LAB was evaluated during 4 weeks of CCN storage.

### 2.6. Determination of the TPC Content and Antioxidant Activity of Prepared CCN

The TPC content was determined by the spectrophotometry method according to the procedure described by Vaher et al. [19].

The ability of the CCN extract to scavenge DPPH free radicals was assessed by the standard method described by Zhu et al. [20].

### 2.7. Statistical Analysis

The results were expressed as the mean ± standard deviation (SD). Preparation of CCN was performed once; all analyses were performed in triplicate. Results were analysed using the statistical package SPSS for Windows V15.0 (SPSS Inc., Chicago, IL, USA, 2007). The significance of differences between the samples was evaluated using Tukey range tests at a 5% level. A linear Pearson’s correlation was used to quantify the strength of the relationship between the variables. The results were recognized as statistically significant at *p* ≤ 0.05. In order to evaluate an influence of different factors (different ingredients used and their interaction) on analyzed parameters of the CCN, Multivariate analysis of variance (ANOVA) was performed and the Tukey HSD test as post-hoc test (statistical program R 3.2.1, R Core Team 2015).

## 3. Results and Discussion

### 3.1. Overall Acceptability (OA) and Emotions (EM) Induced in Consumers by the Prepared Fermented Milk Permeate (MP), Psyllium Husk (Ph), and Apple By-Product Combinations, and CCN

Overall acceptability (OA) and EM induced in consumers by the prepared fermented MP, Ph, and apple by-product combinations are shown in Table 2. By increasing the Ph content in fermented MP, OA of the combinations was increased, and the highest OA (score 9.0) was established for the combination prepared from 100 mL of MP and 7 g of Ph. Similar tendencies were found for the addition of apple by-products: by increasing their quantity in combination with MP and Ph, OA of the combination was increased, and the highest OA (score 8.3) was shown for the combination with 100 mL of fermented MP, 7 g of Ph, and 7 g of apple by-products. Correlation coefficients between the OA and EM induced in consumers by the prepared fermented MP, Ph, and apple by-product combinations are shown in Supplementary file S3. A very strong positive correlation was established between OA and the EM happy” (*r* = 0.834). Moreover, significant negative moderate correlations were found between OA and the EMs “neutral” and “angry” (*r* = −0.594 and *r* = −0.558, respectively).

Overall acceptability (OA) and EMs induced in consumers by prepared CCN are shown in Table 3. The highest OA (score 8.5) was shown for samples whose texture was formed with Gel, and the formula consisted of fermented MP, Ph, apple by-products, cit, and Xy (Gel+MP+Ph+App+cit+Xy). The above-mentioned combination, but without Xy, showed slightly lower OA (score 7.3), and the absence of apple by-products (Gel+MP+Ph+cit) reduced OA of the CCN to a score of 5.4. Comparing CCN groups prepared with agar, similar tendencies were found as in groups prepared with Gel: by adding apple by-products and Xyl, OA of the CCN samples was increased by 34.1% and 53.7%, respectively. However, it should be mentioned that CCN prepared with Gel, in all cases, had a higher OA than those prepared with agar.

Comparing EMs induced in consumers by prepared CNN, the highest EM “happy” was fixed when consumers were testing the CCN with the highest OA CCN, and a very strong positive correlation was found between OA and the EM “happy” (*r* = 0.907 **) (Supplementary file S4). Moreover, moderate positive correlations were found between OA and “scared” (*r* = 0.535 *), as well as between OA and “sad” (*r* = −0.543 **). The highest expression of the EM “neutral” was fixed by testing the Agar+MP+Ph+AA group of CCN; testing of this group also led to lowest expression of the EM “happy” and the highest expression of “sad”, “angry”, and “contempt”. The lowest intensity of the EM “neutral” was fixed when consumers were testing Gel+MP+Ph+cit and Agar+MP+Ph+App+AA+Xy groups of CCN; however, testing of Gel+MP+Ph+cit led to the highest expression of “disgusted” and the lowest expression of “happy”, the opposite to the findings for Agar+MP+Ph+App+AA+Xy, testing of which led to the lowest expression of most of the negative EMs (“sad” and “disgusted”).

EMs are important factors in consumer food choices, and the foods consumed can induce a variety of EMs [21,22,23]. Moreover, it should be pointed out that limited facial movement can be related to a less sensitive measurement of expression of the EMs “sad” and “angry” [24,25]. The EMs “contempt” and “disgusted” belong to the hostile EMs [26], and facial EM expressions can recognize and differentiate the basic tastes and odours [27]. Moreover, consumers express negative expressions more accurately than positive ones [28]. Facial expressions can elucidate the consumer OA of products based on emotional responses and familiarity [16]. In this study, ingredient combinations and CCN were selected as a model of sustainable higher value CCN materials and products for evaluating the hedonic and emotional responses of consumers. The measurements of EM expression showed that the positive EMs (especially “happy”) were more intense than negative ones (e.g., “angry”). These results are in line with other published studies, where it has been described that consumers use more positive EMs to describe foods than negative ones [29,30]. Smiling, in general, is the expression of happiness [31]. However, according to other authors, a smile can be a signal of embarrassment [32] or disappointment [33] or used deliberately to hide EMs [34]. However, in our study, there was a very strong correlation between OA results using a hedonic scale and expression of the EM “happy”. Explicit methods show the conscious and cognitive actions or associations with the food product [21], whereas implicit methods measure the unconscious responses to the stimuli [35]. The results of our study are in agreement with other studies in which correlations were found between results for explicit and implicit measurements [24].

### 3.2. LAB Count in CCN Samples during Storage

**Figure 2:**
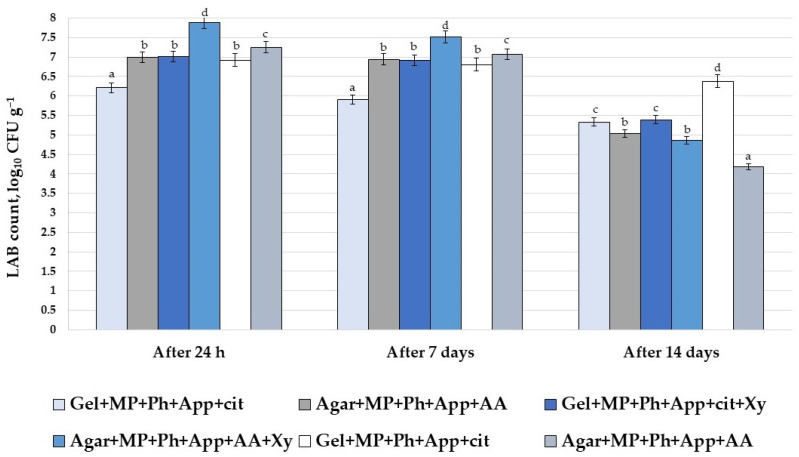
Viable lactic acid bacteria (LAB) count in prepared nutraceutical chewing candy during storage (LAB—lactic acid bacteria; CFU—colony forming units; Gel—with gelatin; Agar—with agar; MP—with fermented milk permeate; Ph—with psyllium husk; cit—with citric acid; AA—with ascorbic acid; Xy—with xylitol; App—with apple by-products. Data are expressed as mean values (n = 3) ± standard deviation (SD); ^a–d^ means within columns with different letters are significantly different, when *p* ≤ 0.05).

The LAB count in CCN samples, after 24 h and 7 and 14 days of storage, is presented in Figure 2. Comparing the LAB count in samples after 24 h, the highest was found in Agar+MP+Ph+App+AA samples (7.88 log_10_ CFU g^−1^); however, after 14 days of storage, these samples showed one of the largest reductions in valuable LAB, compared with other groups (reduced by 1.6 times). Moreover, a LAB count higher than 7.0 log_10_ CFU g^−1^ was established in Agar+MP+Ph+App+AA samples after 24 h; however, after 14 days of storage, the LAB count in these samples reduced by 1.7 times. Comparing the LAB count in all samples after 7 days of storage, in most samples it was higher than 6.0 log_10_ CFU g^−1^ (except Gel+MP+Ph+App+cit samples); this finding is very important, as food containing more than 6.0 log_10_ CFU g^−1^ can have probiotic properties. However, after 14 days of storage, just one sample group showed a LAB count higher than 6.0 log_10_ CFU g^−1^ (Gel+MP+Ph+App+cit).

Incorporation of LAB into functional foods has become very popular, because of their important role in the formation of desirable specific food sensory properties [36] and biosafety [37]. However, LAB are sensitive to environmental conditions [38]. Agar-based gels are used for LAB immobilization, to prolong their viability during storage [39]. Moreover, the presence of pectin in food matrices can prolong LAB viability [40], and the specific characteristics of Ph can lead to a higher LAB count during storage [41]. Multivariate analysis of variance showed that the selection of Gel or agar was a significant factor in the LAB count in CCN samples after 24 h and 7 and 14 days (*p* ≤ 0.0001), the use of apple by-products was a significant factor in the LAB count in CCN samples after 24 h and 7 days (*p* ≤ 0.0001), and the use of Xy was a significant factor in the LAB count in CCN samples after 24 h and 7 days (*p* = 0.002 and *p* = 0.012, respectively). Furthermore, the interaction Gel/agar × apple by-products was significant in the LAB count in CCN samples after 7 days (*p* = 0.04) and the interaction Gel/agar × Xy was a significant factor in the LAB count in CCN samples after 24 h and 14 days of storage (*p* = 0.014 and *p* ≤ 0.0001, respectively). It was reported that Xy inhibits lactobacilli [42], and can be recommended for oral health [43] but, in food formulations, Xy can reduce the viability of LAB. However, to prolong LAB viability in food matrices, Gel can be incorporated [44,45].

### 3.3. CCN Colour Coordinates and Texture

Colour coordinates (L*—lightness, a*—redness, b*—yellowness) and texture parameters of the prepared CCN are shown in Table 4. All the samples showed similar L* values (on average 53.5 ± 3.7 NBS); however, significant differences were established between samples a* and b* coordinates. The lowest a* values were found for CCN groups Gel+MP+Ph+cit (0.12 NBS) and Agar+MP+Ph+AA (0.22 NBS), on average 62.1 times lower than for other sample groups; these changes could be associated with the addition of apple by-products to the CCN formulation. Similar tendencies were found for CCN samples’ b* coordinates, as the addition of apple by-products increased the b* values, the highest being found in the Agar+MP+Ph+App+AA+Xy group (23.3 NBS). Multivariate analysis of variance showed that the selection of Gel or agar, and the use of apple by-products and Xy were significant factors in CCN samples’ a* and b* coordinates (*p* ≤ 0.05). Moreover, the interaction Gel/agar × apple by-products was significant in CCN a* and b* coordinates (*p* ≤ 0.0001 and *p* = 0.003, respectively), and the interaction Gel/agar × Xy was significant in CCN a* coordinates (*p* ≤ 0.0001).

The colour of food can be a key factor in quality perception by consumers, greatly affecting sensory acceptance [46]. Our results are in agreement with Martins et al. [47], who reported that the colour of jelly candies enriched with apple purée got darker during the thermal treatment; this may be as a result of Maillard and caramelization reactions at high temperatures, among other reasons. Fibre (fruit pulp)-enriched candies have a darker colour [48]. Moreover, our results are in agreement with Šeremet et al. [49], who reported that in all cases, a harder texture of samples can be obtained by using Gel rather than agar (by 69.6%, 72.0%, and 46.4%, between groups Gel+MP+Ph+cit and Agar+MP+Ph+AA, Gel+MP+Ph+App+cit and Agar+MP+Ph+App+AA, and Gel+MP+Ph+App+cit+Xy and Agar+MP+Ph+App+AA+Xy, respectively). Increasing the amount of Gel in candy formulations increases hardness, chewiness, and cohesiveness, while decreasing adhesiveness [50]. Addition of apple by-products in the CCN group with Gel increased hardness; however, in the CNN prepared with agar and Xy, it reduced the hardness of the CCN samples. Altınok et al. [51] reported that the particle size of the plant by-product is a significant factor in the texture parameters, especially the hardness, adhesiveness, chewiness, and resilience of gummy candies. Multivariate analysis of variance showed that the selection of Gel or agar was a significant factor in CCN texture (*p* ≤ 0.0001); also, the interaction Gel/agar × Xy was a significant factor in CCN texture (*p* = 0.003).

### 3.4. TPC Content and Antioxidant Activity of Prepared CCN

**Figure 3:**
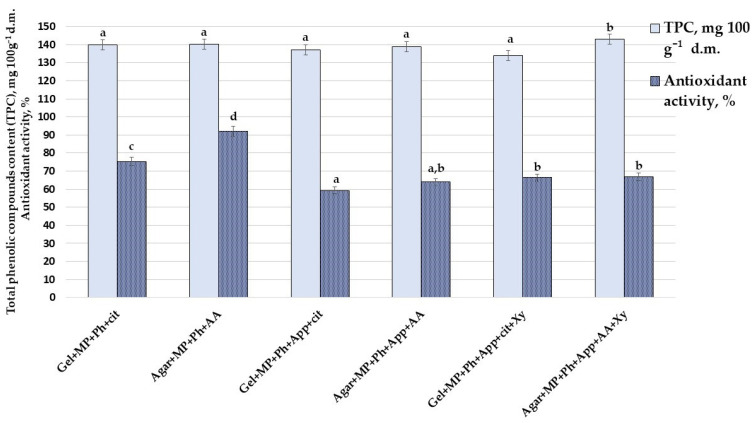
Total phenolic compound (TPC) content (mg 100 g^−1^ d.m.) and antioxidant activity (%) of prepared nutraceutical chewing candy (Gel—with gelatin; Agar—with agar; MP—with fermented milk permeate; Ph—with psyllium husk; cit—with citric acid; AA—with ascorbic acid; Xy—with xylitol; App—with apple by-products. Data expressed as mean values (n = 3) ± standard deviation (SD); ^a–d^ means within columns with different letters are significantly different, when *p* ≤ 0.05).

The development of confectionery products based exclusively on natural ingredients with antioxidant properties may offer valuable solutions to the confectionery industry. The TPC content (mg 100 g^−1^ d.m.) and antioxidant activity (%) of the prepared CCN are shown in Figure 3. The highest TPC content was found in Agar+MP+Ph+App+AA+Xy samples (143.1 mg 100 g^−1^ d.m.); other samples showed, on average, a 3.5% lower TPC content. Different tendencies were found for the antioxidant activity of the CCN, the lowest being found in Gel+MP+Ph+App+cit and Agar+MP+Ph+App+AA samples (59.3% and 63.9%, respectively). The highest antioxidant activity was found in Agar+MP+Ph+App+AA samples (92.0%); that in Gel+MP+Ph+App+cit+Xy and Agar+MP+Ph+App+AA+Xy samples was, on average, 66.6%. The addition of apple by-products and Xy significantly decreased the antioxidant activity (by %) in CCN samples with agar. The same tendency was observed in CCN samples with gelatin.

Fruit products (juices and purées) may provide functional and organoleptic properties in jelly candies in a natural way [52]. In addition, cit can attribute a decline in browning index, increase in acidic taste, and changes in some textural properties (hardness, chewiness, and springiness) of the candies; this is related to the characteristics of the physical properties of H^+^ ions and calcium cations to form gels. On the other side, adding AA, as an active antioxidant compound, leads to elevated antioxidant capacity, lowered browning index, and the lowest rates of electrolyte leakage [53]. The decrease of TPC can be related to polyphenol–dietary fibre non-covalent binding including hydrogen bonds, van der Waals forces, and hydrophobic interactions [54], resulting in changes of polyphenol activity, bioaccessibility, and bioavailability.

Multivariate analysis of variance showed that the selection of Gel or agar, and the use of apple by-products and Xy were significant factors in CCN antioxidant activity (*p* ≤ 0.001, *p* ≤ 0.0001, and *p* = 0.025, respectively); also, the interaction Gel/agar × apple by-products was a significant factor in CCN antioxidant activity (*p* = 0.01).

## 4. Conclusions

The highest OA (score 8.5) was shown for samples consisting of MP, Ph, apple by-products, cit and Xy; a very strong correlation was found between OA and the EM “happy” (*r* = 0.907 **). After 14 days of storage, Gel+MP+Ph+App+cit samples showed a LAB count higher than 6.0 log_10_ CFU g^−1^; however, better antioxidant properties were found for the CCN prepared with agar. Finally, it can be stated that fermented MP, Ph, and apple by-products can be used for preparation of added-value CCN in a sustainable manner, and the recommended formulation is Gel+MP+Ph+App+cit+Xy.

## Figures and Tables

**Table 1:** Nutraceutical chewing candy (CCN) formulas.

Nutraceutical Chewing Candy Formula	Milk Permeate	Psyllium Husk	Apple By-Products	Water	Gelatin	Agar	Citric Acid	Ascorbic Acid	Xylitol
	mL	g		mL	g				
Gel+MP+Ph+cit	100	7	-	100	10	-	1	-	-
Agar+MP+Ph+AA			-		-	10	-	1	-
Gel+MP+Ph+App+cit			7		10	-	1	-	-
Agar+MP+Ph+App+AA					-	10	-	1	-
Gel+MP+Ph+App+cit+Xy					10	-	1	-	4
Agar+MP+Ph+App+AA+Xy					-	10	-	1	4

Gel—with gelatin; Agar—with agar; MP—with fermented milk permeate; Ph—with psyllium husk; cit—with citric acid; AA—with ascorbic acid; Xy—with xylitol; App—with apple by-products.

**Table 2:** Overall acceptability (OA) and emotions (EMs) induced in consumers by the prepared fermented milk permeate (MP), psyllium husk (Ph), and apple by-product combinations.

Ingredient Combination	OA	Emotions Induced in Consumers (from 0 to 1)								
		Neutral	Happy	Sad	Angry	Surprised	Scared	Disgusted	Contempt	Valence
MP+3gPh	5.7± 0.3 a	0.42 ± 0.03 b	0.14 ± 0.02 b	0.09 ± 0.01 a	0.05 ± 0.01 b	0.002 ± 0.0003 a	0.003 ± 0.0005 b	0.001 ± 0.0003 a	0.01 ± 0.003 a	0.104 ± 0.011 a
MP+5gPh	7.7 ± 0.5 b	0.44 ± 0.02 b	0.17 ± 0.01 b	0.17 ± 0.02c	0.01 ± 0.001 a	0.007 ± 0.001c	0.001 ± 0.0004 a	0.001 ± 0.0004 a	0.03 ± 0.001 c	0.107 ± 0.009 a
MP+7gPh	9.0 ± 0.4c	0.24 ± 0.01 a	0.33 ± 0.02d	0.07 ± 0.01 a	0.02 ± 0.002 a	0.001 ± 0.0002 a	0.002 ± 0.0006 a, b	0.001 ± 0.0003 a	0.09 ± 0.004 f	0.114 ± 0.008 a
MP+7gPh+2.5gApp	5.7 ± 0.5 a	0.49 ± 0.04 b	0.13 ± 0.02 b	0.11 ± 0.01 b	0.04 ± 0.003 b	0.010 ± 0.0002 d	0.001 ± 0.0003 a	0.002 ± 0.0006 b	0.07 ± 0.002 e	0.110 ± 0.009 a
MP+7gPh+5.0gApp	6.8 ± 0.4 b	0.65 ± 0.05c	0.09 ± 0.01 a	0.13 ± 0.02 b	0.08 ± 0.004c	0.003 ± 0.0004 a, b	0.001 ± 0.0004 a	0.001 ± 0.0003 a	0.02 ± 0.001 b	0.121 ± 0.010 a
MP+7gPh+7.0gApp	8.3 ± 0.4c	0.21 ± 0.02 a	0.23 ± 0.03 c	0.08 ± 0.01 a	0.01 ± 0.003 a	0.002 ± 0.0003 a	0.001 ± 0.0003 a	0.001 ± 0.0004 a	0.04 ± 0.003 d	0.113 ± 0.011 a

OA—overall acceptability; MP—milk permeate; Ph—psyllium husk; App—apple by-products. a–f Means within a row with different letters are significantly different (*p* ≤ 0.05).

**Table 3:** Overall acceptability (OA) and emotions (EMs) induced in consumers by the prepared nutraceutical chewing candy (CCN).

CCN	OA	Emotions Induced in Consumers (from 0 to 1)								
		Neutral	Happy	Sad	Angry	Surprised	Scared	Disgusted	Contempt	Valence
Gel+MP+Ph+cit	5.4 ± 0.4 b	0.33 ± 0.03 a	0.12 ± 0.02 a	0.20 ± 0.03 b	0.002 ± 0.001 b	0.005 ± 0.0002 c	0.014 ± 0.002 c	0.019 ± 0.004 d	0.08 ± 0.01 b	0.43 ± 0.05 a
Agar+MP+Ph+AA	4.1 ± 0.3 a	0.64 ± 0.05 d	0.11 ± 0.02 a	0.44 ± 0.04 c	0.007 ± 0.001 e	0.008 ± 0.001 d	0.002 ± 0.0001 b	0.004 ± 0.001 b	0.23 ± 0.03 d	0.54 ± 0.04 b
Gel+MP+Ph+App+cit	7.3 ± 0.5 c	0.52 ± 0.04 c	0.31 ± 0.03 c	0.20 ± 0.05 b	0.001 ± 0.0003 a	0.013 ± 0.002 e	0.030 ± 0.005 d	0.003 ± 0.001 b	0.01 ± 0.002 a	0.53 ± 0.03 b
Agar+MP+Ph+App+AA	5.5 ± 0.4 b	0.47 ± 0.05 b	0.13 ± 0.02 a	0.11 ± 0.02 a	0.003 ± 0.0005 c	0.009 ± 0.001 d	0.001 ± 0.0005 a	0.010 ± 0.002 c	0.08 ± 0.01 b	0.58 ± 0.04 b
Gel+MP+Ph+App+cit+Xy	8.5 ± 0.6 d	0.41 ± 0.04 b	0.28 ± 0.03 c	0.13 ± 0.03 a	0.004 ± 0.0003 d	0.003 ± 0.0005 b	0.012 ± 0.002 c	0.020 ± 0.005 d	0.15 ± 0.03 c	0.41 ± 0.03 a
Agar+MP+Ph+App+AA+Xy	6.3 ± 0.2 c	0.29 ± 0.03 a	0.19 ± 0.02 b	0.16 ± 0.04 a	0.002 ± 0.0004 b	0.001 ± 0.0005 a	0.002 ± 0.0006 b	0.001 ± 0.0003 a	0.07 ± 0.01 b	0.69 ± 0.05 c

CCN—nutraceutical chewing candy; OA—overall acceptability; Gel—with gelatin; Agar—with agar; MP—with fermented milk permeate; Ph—with psyllium husk; cit—with citric acid; AA—with ascorbic acid; Xy—with xylitol; App—with apple by-products. a–e Means within a row with different letters are significantly different (*p* ≤ 0.05).

**Table 4:** Colour coordinates and texture parameters of the prepared CCN.

CCN	Colour Coordinates, NBS			Texture, mJ
	L*	a*	b*	
Gel+MP+Ph+cit	54.4 ± 3.5 a	0.12 ± 0.02 a	18.2 ± 0.5 a	2.3 ± 0.2c
Agar+MP+Ph+AA	56.8 ± 2.9 a	0.22 ± 0.02 b	20.8 ± 0.6 b	1.6 ± 0.1 b
Gel+MP+Ph+App+cit	49.9 ± 3.4 a	5.93 ± 0.21 e	22.5 ± 0.4 c	2.5 ± 0.2 c, d
Agar+MP+Ph+App+AA	53.3 ± 4.0 a	5.05 ± 0.14 d	22.9 ± 0.5 c	1.8 ± 0.1 b
Gel+MP+Ph+App+cit+Xy	51.5 ± 2.9 a	6.01 ± 0.27 f	22.4 ± 0.6 c	2.8 ± 0.3 d
Agar+MP+Ph+App+AA+Xy	54.9 ± 3.5 a	4.11 ± 0.23 c	23.3 ± 0.4 d	1.3 ± 0.1 a

L*—lightness; a*—redness/greenness; b*—yellowness/blueness); CCN—nutraceutical chewing candy; Gel—with gelatin; Agar—with agar; MP—with fermented milk permeate; Ph—with psyllium husk; cit—with citric acid; AA—with ascorbic acid; Xy—with xylitol; App—with apple by-products. Data expressed as mean values (n = 3) ± standard deviation (SD). a–f Means within a row with different letters are significantly different, when *p* ≤ 0.05.

## Data Availability

The data are available from the corresponding author, upon reasonable request.

## Supplementary Materials

The following are available online at https://www.mdpi.com/article/10.3390/foods10040777/s1, Supplementary File S1 Characteristics of permeate: Table S1. Parameters after 48 h of milk permeate fermented with LUHS29 strain, Table S2. Antimicrobial properties; Supplementary File S2 Antimicrobial properties of apple by-products: Table S3. Antimicrobial properties of apple by-products. Supplementary File S3. Table S4. Correlation coefficients between overall acceptability and emotions induced in consumers by the prepared fermented milk permeate, psyllium husk, and apple by-product combinations. Supplementary File S4. Table S5. Correlation coefficients between overall acceptability and emotions induced in consumers by the prepared nutraceutical chewing candy.
