# Supplementary material for: Characteristics of Nutraceutical Chewing Candy Formulations Based on Fermented Milk Permeate, Psyllium Husk, and Apple By-Products
Source: Foods. 2021 Apr 5;10(4):777. doi: 10.3390/foods10040777 (PMC8065903; doi:10.3390/foods10040777)
Supplement: Supplementary file 1 [file foods-10-00777-s001.zip › Supplementary file S1. Characteristics of permeate.docx]

**Table S1.** Parameters after 48 h of milk permeate fermented with LUHS29 strain.

| **Milk permeate samples** | **pH** | | **TTA, °N** | | **LAB count,**  **log_10_ CFU mL^-1^** | | **Lactose, g 100g^-1^** | | **GOS mg 100 mL^-1^** | |
| --- | --- | --- | --- | --- | --- | --- | --- | --- | --- | --- |
|  |  |  |  |  |  |  |  |  | **G2** | **G3** |
| **MP_NF_** | 5.88 ± 0.80b | | 3.00 ± 0.14a | | nd | | 10.48 ± 0.28b | | nd | nd |
| **MP_LUHS29_** | 3.91 ± 0.23a | | 9.50 ± 0.19b | | 8.19 ± 0.23 | | 5.05 ± 0.19a | | 21.70 ± 0.33 | 5.10 ± 0.11 |
|  | **Overall**  **accep-tability** | **Emotions induced by the beverages (from 0 to 1)** | | | | | | | | |
|  |  | **Neutral** | **Happy** | **Sad** | **Angry** | **Surprised** | **Scared** | **Disgusted** | **Contempt** | **Valence** |
| MP_NF_ | 5.20  ± 0.18 | 0.370  ± 0.020b | 0.130  ± 0.003 | 0.180  ± 0.004b | 0.060  ± 0.001a | 0.030  ± 0.001b | 0.0010  ± 0.00002 | 0.00100  ± 0.00002 | 0.0900  ± 0.0020b | 0.080  ± 0.002a |
| MP_LUHS29_ | 5.30  ± 0.13 | 0.230 ± 0.004a | 0.14  ± 0.003 | 0.160  ± 0.003a | 0.130  ± 0.003b | 0.010  ± 0.0002a | 0.00100  ± 0.00002 | 0.00100  ± 0.00002 | 0.0300  ± 0.0006a | 0.130  ± 0.003b |
| LAB, lactic acid bacteria; CFU, colony-forming units; TTA, total titratable acidity; G2, galactobiose; G3, galactotriose;  MP, milk permeate; MP_LUHS29_, fermented with LUHS29 (*P. acidilactici);* MP_NF_, unfermented; GOS, galactooligosaccharides.  Data are represented as means (n = 3) ± SD. ^a-b^ Means with different letters in column are significantly different (*p* ≤0.05). | | | | | | | | | | |

**Table S2.** The diameter of inhibition zones (mm) of the prepared beverages against pathogenic and opportunistic strains of milk permeate fermented with LUHS29 strain.

| **Samples** | **Diameter of Inhibition Zones (DIZ) (mm)** | | | | | | | | | | | | | | |
| --- | --- | --- | --- | --- | --- | --- | --- | --- | --- | --- | --- | --- | --- | --- | --- |
|  | **Pathogenic and opportunistic bacteria strains** | | | | | | | | | | | | | | |
|  | **1** | **2** | **3** | **4** | **5** | **6** | **7** | **8** | **9** | **10** | **11** | **12** | **13** | **14** | **15** |
| MP_LUHS29_ | nd | nd | nd | nd | nd | nd | nd | nd | n.d | 12.7  ± 0.4 | nd | nd | nd | nd | 15.0  ± 0.1 |
| MP_NF_ | nd | nd | nd | nd | nd | nd | nd | nd | nd | nd | nd | nd | nd | nd | nd |
| MP, milk permeate; MP_LUHS29_, fermented with LUHS29 (*P. acidilactici*); MP_NF_, unfermented; nd – not determined; 1, *Klebsiella pneumonia*; 2, *Salmonella enterica*; 3, *Pseudomonas aeruginosa*; 4, *Acinetobacter baumannii*; 5, *Proteus mirabilis*, 6, MRSA M87fox; 7, *Enterococcus faecalis*; 8, *Enterococcus faecium*; 9, *Bacillus cereus*; 10, *Streptococcus mutans*; 11, *Enterobacter cloacae*; 12, *Citrobacter freundii*; 13, *Streptococcus epidermis*, 14, *Staphylococcus haemolyticus*; 15, *Pasteurella multocida*.  Data are represented as means (n = 3) ± SD. | | | | | | | | | | | | | | | |
