# Supplementary material for: Characteristics of Nutraceutical Chewing Candy Formulations Based on Fermented Milk Permeate, Psyllium Husk, and Apple By-Products
Source: Foods. 2021 Apr 5;10(4):777. doi: 10.3390/foods10040777 (PMC8065903; doi:10.3390/foods10040777)
Supplement: Supplementary file 1 [file foods-10-00777-s001.zip › Supplementary file S2. Antimicrobial activity of apple by-products.docx]

**Table S3.** Antimicrobial activity of the apple by-products.

| B/F sam-ples | Inhibition zones, mm | | | | | | | | | | | | | | |
| --- | --- | --- | --- | --- | --- | --- | --- | --- | --- | --- | --- | --- | --- | --- | --- |
|  | Pathogenic and opportunistic bacteria strains | | | | | | | | | | | | | | |
|  | *Klebsiella pneumoniae* | *Salmonella enterica* 24 SPn06 | *Pseudomonas aeruginosa* 17-331 | *Acinetobacter baumannii* 17-380 | *Proteus mirabilis* | *MRSA* M87fox | *Enterococcus faecalis* 86 | *Enterococcus faecium* 103 | *Bacillus cereus* 18 01 | *Streptococcus mutans* | *Enterobacter cloacae* | *Citrobacter freundii* | *Streptococcus epidermis* | *Staphylococcus haemolyticus* | *Pasteurella multocida* |
| Appl _vacuum_ | - | - | - | - | - | - | - | - | 12.2  ±0.3 | - | - | - | 10.3  ±0.2 | 11.5  ±0.3 | 21.3  ±0.4 |
| Data expressed as a mean values (n = 3) ± SD; SD – standard deviation.  MRSA – Methicillin-resistant *Staphylococcus aureus*; Appl – apples; vacuum - vacuum dried. | | | | | | | | | | | | | | | |
