# Supplementary material for: Characteristics of Nutraceutical Chewing Candy Formulations Based on Fermented Milk Permeate, Psyllium Husk, and Apple By-Products
Source: Foods. 2021 Apr 5;10(4):777. doi: 10.3390/foods10040777 (PMC8065903; doi:10.3390/foods10040777)
Supplement: Supplementary file 1 [file foods-10-00777-s001.zip › Supplementary file S3. Correlation coefficients between OA and emotions by the prepared combinations.docx]

**Table S4.** Correlation coefficients between overall acceptability and emotions induced in consumers by the prepared fermented milk permeate, psyllium husk, and apple by-product combinations.

|  | | **Emotions induced in consumers** | | | | | | | | |
| --- | --- | --- | --- | --- | --- | --- | --- | --- | --- | --- |
|  | | **Neutral** | **Happy** | **Sad** | **Angry** | **Surprised** | **Scared** | **Disgusted** | **Contempt** | **Valence** |
| OA | r | −0.594** | 0.834** | −0.114 | −0.558* | −0.407 | −0.087 | −0.224 | 0.442 | 0.387 |
|  | p | 0.009 | 0.0001 | 0.652 | 0.016 | 0.093 | 0.732 | 0.371 | 0.066 | 0.113 |
| OA – overall acceptability; r – Pearson correlation; p – significance; ** – correlation is significant at the 0.01 level (2-tailed);  * – correlation is significant at the 0.05 level (2-tailed). | | | | | | | | | | |
