# Supplementary material for: Characteristics of Nutraceutical Chewing Candy Formulations Based on Fermented Milk Permeate, Psyllium Husk, and Apple By-Products
Source: Foods. 2021 Apr 5;10(4):777. doi: 10.3390/foods10040777 (PMC8065903; doi:10.3390/foods10040777)
Supplement: Supplementary file 1 [file foods-10-00777-s001.zip › Supplementary file S4. Correlation coefficients between OA and emotions induced in consumers by the prepared CCN.docx]

**Table S5.** Correlation coefficients between overall acceptability and emotions induced in consumers by the prepared nutraceutical chewing candy.

|  | | **Emotions induced in consumers** | | | | | | | | |
| --- | --- | --- | --- | --- | --- | --- | --- | --- | --- | --- |
|  | | **Neutral** | **Happy** | **Sad** | **Angry** | **Surprised** | **Scared** | **Disgusted** | **Contempt** | **Valence** |
| OA | r | −0.259 | **0.907**** | **−0.543*** | −0.373 | −0.100 | **0.535*** | 0.344 | −0.322 | −0.189 |
|  | p | 0.300 | **0.0001** | **0.020** | 0.128 | 0.692 | **0.022** | 0.162 | 0.192 | 0.452 |
| OA – overall acceptability; r – Pearson correlation; ** – correlation is significant at the 0.01 level (2-tailed); * – correlation is significant at the 0.05 level (2-tailed). | | | | | | | | | | |
